# Glutamate Ionotropic Receptor Kainate Type Subunit 3 (GRIK3) promotes epithelial‐mesenchymal transition in breast cancer cells by regulating SPDEF/CDH1 signaling

**DOI:** 10.1002/mc.23014

**Published:** 2019-04-11

**Authors:** Bin Xiao, Zhenzhan Kuang, Weiyun Zhang, Jianfeng Hang, Lidan Chen, Ting Lei, Yongyin He, Chun Deng, Weiwei Li, Jingrun Lu, Jing Qu, Quan Zhou, Wenbo Hao, Zhaohui Sun, Linhai Li

**Affiliations:** ^1^ Department of Laboratory Medicine General Hospital of Southern Theatre Command of PLA Guangzhou China; ^2^ Department of Basic Clinical Laboratory Medicine, School of Clinical Laboratory Science Guizhou Medical University Guiyang China; ^3^ Department of Laboratory Medicine Nanfang Hospital, Southern Medical University Guangzhou China; ^4^ Institute of Antibody Engineering, School of Laboratory Medicine and Biotechnology Southern Medical University Guangzhou China

**Keywords:** breast cancer, CDH1, epithelial‐mesenchymal transition (EMT), glutamate ionotropic receptor kainate type subunit 3 (GRIK3)

## Abstract

Glutamate Ionotropic Receptor Kainate Type Subunit 3 (GRIK3) is an important excitatory neurotransmitter receptor that plays a significant role in various neurodegenerative diseases. However, the biological functions of GRIK3 in malignancies are largely unknown because of limited related studies. Here, we primarily reported that the expression of GRIK3 was higher in breast cancer tissues than in adjacent noncancerous tissues. GRIK3 expression was also positively correlated with the prognosis of patients with breast cancer. GRIK3 promoted the proliferation and migration abilities of breast cancer cells and enhanced the growth of orthotopically implanted tumors. Mechanically, GRIK3 influenced a range of signaling pathways and key signal transducers, including two epithelial‐mesenchymal transition regulators, SPDEF and CDH1. Heterogenous expression of SPDEF and CDH1 counteracted the migration and invasion abilities, respectively, of breast cancer cells induced by GRIK3. Moreover, overexpression of GRIK3 increased the expression of mesenchymal markers and decreased the expression of epithelial markers, resulting in the translocation of β‐catenin into the nucleus and the increased β‐catenin transcriptional activity. In conclusion, the present study reported a novel oncogenic role of GRIK3. Meanwhile, GRIK3, as a membrane receptor, may also serve as a potential therapeutic target for the treatment of breast cancer.

## 1 INTRODUCTION

Breast cancer is one of the most serious threats to the health of women and is the leading disease in gynecological oncology. However, current conventional therapeutic targets for breast cancer treatment are still limited and drug resistance has become a growing problem. Therefore, developing novel therapeutic targets and designing targeted drugs will greatly improve the clinical treatment of breast cancer.

Ionotropic glutamate receptors (iGluRs) serve as the first messenger of glutamic acid through binding to glutamic acid and mediating the signal transmission. iGluRs are divided into three subfamilies on the basis of the structural similarity: N‐methyl‐d‐aspartate (NMDA), alpha‐amino‐3‐hydroxy‐5‐methyl‐4‐iso‐xazolepropionic acid and kainate receptors (KA‐R).^1^ Glutamate receptor ionotropic, kainate 3 (GRIK3) is the member of the ionotropic glutamate KA‐R subfamily, a branch of glutamate receptor family, which plays a critical role in synaptic potentiation—an essential process for learning and memory.^2^, ^3^, ^4^, ^5^, ^6^ To our knowledge, few studies have reported on the association of GRIK3 with cancer. The expression of GRIK3 was found in rhabdosarcoma, neuroblastoma, thyroid tumor, lung cancer, breast cancer, astrocytoma, multiple myeloma, glioma, and colorectal cancer.^7^ Moreover, the GRIK3 gene was found to be methylated across all stages of lung adenocarcinoma, indicating that GRIK3 might be an epigenetic marker for diagnosis.^8^ Recently, Gong et al^9^ showed that GRIK3 expression could serve as an independent prognostic biomarker and a novel treatment target for patients with gastric cancer. However, the precise mechanism by which GRIK3 expression influences cancer progression remains unclear.

It has been shown that GRIK3 is enriched in the neuroactive ligand receptor interaction pathway and haploinsufficiency of GRIK3 may be responsible for the severe developmental delay.^2^, ^9^, ^10^ Interestingly, a previous study indicated that, in breast cancer, multiple estradiol (E2) stimulated or inhibited genes also enriched in the neuroactive ligand receptor interaction pathway were able to affect the cell proliferation.^11^ On the basis of the current studies, we proposed that GRIK3 might function in the development of breast cancer.

In the present study, we sought to gain new insights into the role of GRIK3 in the progression and development of breast cancer. Our study aimed to reveal the expression and clinical significance of GRIK3 in breast cancer, explore further whether the overexpression of GRIK3 protein is a key step in the growth and metastasis of breast cancer, and explore the specific molecular mechanism GRIK3 affects breast cancer development.

## 2 MATERIALS AND METHODS

### 2.1 Cell lines and culture condition

Breast cancer cell lines (MCF‐7, MDA‐MB‐231, BT474) were purchased from and authenticated by the Typical Culture Preservation Commission Cell Bank (Chinese Academy of Sciences, Shanghai, China). All cell lines were cultured in Dulbecco modified Eagle medium (Gibco BRL, New York) supplemented with 10% fetal bovine serum (Gibco BRL). All cell lines were cultured at 37°C with 5% CO_2_.

### 2.2 Heat map of transcriptional profile of GRIK family members

The level 3 RNA sequencing (RNA‐seq) data of breast cancer was downloaded from the TCGA database (https://gdc‐portal.nci.nih.gov/). The samples that contain the information of four different molecular subtypes were further screened, including 37 HER2 + breast cancer, 443 Luminal A breast cancer, 126 Luminal B breast cancer, 115 Triple‐negative breast cancer (TNBC) and 76 adjacent nontumor tissues. The messenger RNA (mRNA) expression data of five GRIK family members, from GRIK1 to GRIK5, was extracted and the heat map was drawn using R software pheatmap (https://cran.r‐project.org/web/packages/pheatmap/index.html). The relative expression levels of these genes were shown with a log2‐transformed value.

### 2.3 Immunohistochemistry and scoring

Sections of PFA‐fixed, paraffin‐embedded tumor tissues were de‐waxed and rehydrated by washing with graded ethanol solutions. The tumor sections were immersed in citrate buffer (pH 6.0) and heated in boiling water for 15 minutes for antigen retrieval. Sections were treated with 3% H_2_O_2_ for 10 minutes to quench endogenous peroxidase activity. Five percent goat serum was used to block nonspecific binding. Sections were then incubated with the primary antibody of GRIK3(1:200, Cell Signaling Technology, Danvers, MA) in a humid box at 4°C overnight. After three washes, the sections were incubated with the second antibody for 2 hours. A 3, 3′‐diaminobenzidine tetrahydrochloride Liquid Substrate Kit (Agilent Technologies, Santa Clara, CA) was used to detect the antigen‐antibody complex and hematoxylin (Sigma Diagnostics, St. Louis, MO) was used to counterstain the tumor sections. Immunostaining images were captured under a microscope (Olympus FV1200MPE, Japan) and the intensity of immunostaining was scored by Image‐Pro Plus 6.0 software.

### 2.4 Statistical analysis

The data were presented as mean ± standard error of the mean. All experimental data were analyzed using the SPSS 13.0 (SPSS Inc, Chicago, IL) and Graphpad Prism 6 software. Kaplan‐Meier plots and the log‐rank test were used to construct the survival curve. Paired or independent the Student *t* test was used to compare two groups with Gaussian data. Differences were considered significant when the *P* value was less than 0.05. (*P < 0.05, **P < 0.01 and ***P < 0.001)

### 2.5 Immunofluorescence assay

The cells were seeded on coverslips laid in six‐well plates and cultured at 37°C in 5% CO_2_ for 12 hours. Before staining, the cells were fixed with 4% paraformaldehyde for 30 minutes, washed three times with PBS, and permeabilized through incubation with 0.25% Triton X‐100 (Biyuntian) solution. The cells were incubated with 10% goat serum for 15 minutes at room temperature to block nonspecific binding. The cells were incubated with primary antibodies against GRIK3, E‐cadherin and cadherin (1:100, Cell Signaling Technology) overnight at 4°C. Subsequently, the cells were incubated with Alexa Fluor 488 goat antimouse IgG (Cell Signaling Technology) or Alexa Fluor 588 goat antimouse IgG (Cell Signaling Technology for 1 hour at room temperature. DAPI (Biyuntian, China) was used to stain the cell nuclei. Fluorescence images were captured by a laser‐scanning confocal microscope (Olympus FV1200MPE). The expression levels of proteins determined by the intensity of fluorescence were calculated by Image‐Pro Plus 6.0 software.

### 2.6 Colony formation assay

The colony formation assay was performed in a six‐well plate. The cells were cultured at 37°C in 5% CO_2_ for 15 to 20 days until colonies formed. The cells (colonies) were fixed with methanol and stained with 0.5% crystal violet. The number of colonies was counted three times by three independent laboratory technicians.

### 2.7 Cell proliferation assays

Breast cancer cells were seeded in 96‐well plates (1 × 10^4^cells per well) and cultured at 37°C in 5% CO_2_. At different time points, the cell culture medium was replaced with 10 µl Cell Counting Kit‐8 (CCK‐8; Sigma Diagnostics) solution and 90 µL completed medium and incubated at 37°C for 2 hours. The OD_450_ was measured using a ELx808 microplate reader (BioTek, Winooski, VT).

### 2.8 Mouse experiment

The design and conduct of all experiments using mice were examined and approved by the Institutional Animal Care and Use Committee (IACUC) of Guangzhou General Hospital of Guangzhou Military Command. Mice (4‐6 weeks old, male) were subcutaneously injected with tumor cells (five mice per group) on the flank. The tumor sizes were measured with a vernier caliper 1 week after the inoculation and calculated by the formula of π/3 × 4 (width/2)2 × (length/2). The tumor size did not exceed 10% of normal body weight of each mouse, which followed the IACUC guideline. Meanwhile, the weight of the mice was recorded each week. To analyze the effect of GRIK3 overexpressing breast cancer cells on the survival of nude mice, we prepared another cohort of mice, with six mice subcutaneously injected with GRIK3 overexpressing cells and 12 mice with control cells. The survival time was recorded each week. The masses of tumors and the lifetime of mice in the different groups were compared using the two‐tailed paired the Student *t* test.

### 2.9 Quantitative Real‐Time polymerase chain reaction

RNA was extracted from tissue or cells using TRIzol reagent (Invitrogen, Carlsbad, CA) and complementary DNA was obtained by reverse transcription using the PrimeScrip RT‐PCR kit (Takara, Tokyo), following the manufacturers' protocols. Real‐time PCR was performed on an ABI 7500 RT‐PCR instrument (Applied Biosystems, Singapore). The amplification parameters were as follows: 30 seconds at 95°C, followed by 40 cycles at 95°C for 5 seconds and 65°C for 34 seconds. The melt curve procedure was as follows: 15 seconds at 95°C, followed by 60°C for 1 minute and 95°C for 15 seconds. Primers used for quantitative real‐time polymerase chain reaction (qRT‐PCR) were as follows: GRIK3 forward 5′‐GCTGGTCTGCACTGAACTCT‐3′ and GRIK3 reverse 5′‐AAAGGGCATCCCCTGAATGG‐3′; GAPDH forward 5′‐AGGTGAAGGTCGGAGTCAAC‐3′ and GAPDH reverse 5′‐CGCTCCTGGAAGATGGTGAT‐3′; MMP3 forward 5′‐CGGTTCCGCCTGTCTCAAG‐3′ and MMP3 reverse 5′‐CGCCAAAAGTGCCTGTCTT‐3′; MMP7 forward 5′‐ATGTGGAGTGCCAGATGTTGC‐3′ and MMP7 reverse 5′‐AGCAGTTCCCCATACAACTTTC‐3′; c‐myc forward 5′‐GTCAAGAGGCGAACACACAAC‐3′ and c‐myc reverse 5′‐TTGGACGGACAGGATGTATGC‐3′; cyclin D1 forward 5′‐CAATGACCCCGCACGATTTC‐3′ and cyclin D1 reverse 5′‐CATGGAGGGCGGATTGGAA‐3′.

### 2.10 Transcription sequencing and differentially expressed gene analysis

Total RNA from assay or control cell sample was isolated using TRIzol reagent (Invitrogen) following the manufacturer's instructions. AvBioanalyzer 2100 (Agilent Technologies) and RNA 6000 NanoLabChip Kit (Agilent Technologies) were used to determine the quality and purity of the isolated RNA. The complementary DNA library was constructed by reverse‐transcribing RNA fragments using the mRNA‐Seq sample preparation kit (Illumina, San Diego, CA) and sequenced on an Illumina Hiseq. 4000 (Illumina), using the manufacturer's protocol. The sequencing results underwent bioinformatics analysis and the differentially expressed genes were identified with a cutoff value of log2 (fold change) > 1 or log2 (fold change) < −1 using the R edgeR package. Values of *P* < 0.05 were considered statistically significant.. Gene ontology (GO) enrichment (www.geneontology.org) and KEGG (www.genome.jp/kegg/) pathway analyses were performed to analyze and classify the differentially expressed genes.

### 2.11 Dual‐luciferase reporter assay

The β‐catenin promoter (promoter sequence: −200/0) was cloned into a firefly luciferase reporter gene plasmid, pGL6‐luc to generate pGL6‐β‐catenin‐luc. Three breast cancer cell lines, including the control cells, GRIK3 overexpressing cells and GRIK3 overexpressing cells transfected with siGRIK3‐2, were cultured in 12‐well plates (3 × 10^5^ cells/well) and cotransfected with 2 μg pGL6‐β‐catenin‐luc and 100 ng Renilla luciferase reporter gene plasmid, pRLTK. 24 hours post transfection, the cells were lysed and luciferase activity was measured with the Dual‐Luciferase Reporter Assay System (Cat. E1910. Madison, WI).

## 3 RESULTS

### 3.1 GRIK3 expression is upregulated in breast cancer tissues and breast cancer cell lines

**Figure 1:**
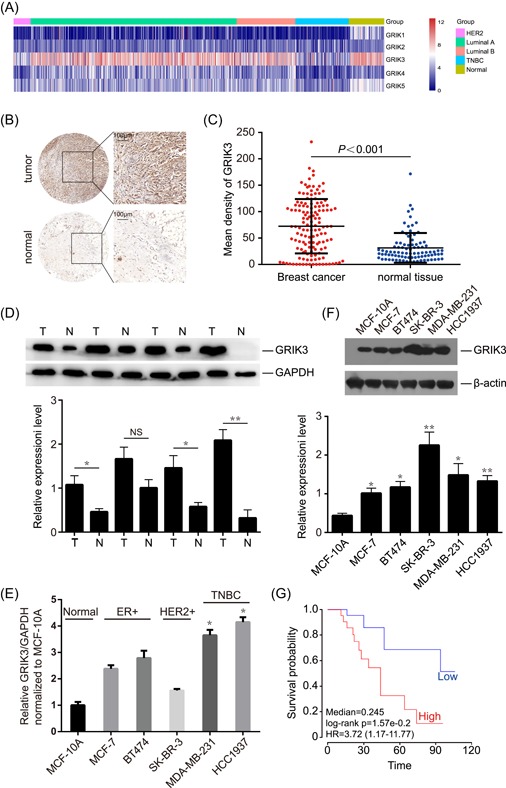
GRIK3 is upregulated in breast cancer tissues and cell lines. A, Transcription profile of GRIK family members in different molecular subtypes of breast cancer. B, Representive immunohistochemistry (IHC) showing the expression of GRIK3 in breast cancer tissues and adjacent normal tissues. C, Mean density of GRIK3 expression was scored in 140 breast cancer tissues and 90 adjacent normal tissues. *P* value was calculated by One‐way ANOVA. D, Western blot analysis showing GRIK3 expression in paired breast tumor and normal tissues. T, Tumor, N, Normal. *P < 0.1 by One‐way ANOVA. NS: No significance. E, The expression of GRIK3 mRNA levels was examined in different breast cancer cell lines. *P < 0.05 by one‐way ANOVA. F, Western blot showing the expression of GRIK3 protein levels in different breast cancer cell lines. *P < 0.05, **P < 0.01 by One‐way ANOVA. G, Kaplan‐Meier analysis of survival probability of breast cancer patients, separated by high and low GRIK3 expression. ANOVA, analysis of variance; GRIK3, glutamate receptor ionotropic, kainate 3; mRNA, messenger RNA [Color figure can be viewed at wileyonlinelibrary.com]

To explore the expression of GRIK family members in breast cancer tissues at mRNA level, we extracted the RNA‐seq data of breast cancer from TCGA and showed the transcription patterns of GRIK family members in different molecular subtypes of breast cancer (Figure 1A). Comparing with normal tissues, the mRNA expression of GRIK3 in HER2+ and TNBC samples was significantly lower (*P*<0.001). Surprisingly, GRIK3 mRNA expression was upregulated in Luminal A and Luminal B samples compared with nontumor tissues (*P* < 0.05). GRIK3 expression was further examined in 140 breast cancer tissues and 90 paired adjacent non‐cancerous tissues using tissue arrays. The expression of GRIK3 at protein level was significantly higher in tumor tissues than in nontumor tissues (*P* < 0.001; Figure 1B and 1C). Furthermore, the total protein was extracted from four paired breast cancer tissues and corresponding adjacent tissues, and GRIK3 expression was examined by Western blot. Protein expression analysis showed similar results, with higher GRIK3 levels in tumor tissue extracts (Figure 1D). The inconsistent results between GRIK3 mRNA level and protein level indicated that GRIK3 might be processed by posttranslational modification in breast cancer. The analysis of GRIK3 mRNA expression in five different breast cancer cell lines with different molecular classification and one normal breast cell line, MCF10A, showed higher GRIK3 mRNA levels in the five breast cancer cell lines. Among the cancerous cell lines, GRIK3 mRNA level was much higher in TNBC and relatively lower in HER2 positive cells (Figure 1E). Kaplan‐Meier analysis using the mean GRIK3 expression score of 52 breast cancer samples as a cutoff point revealed that higher GRIK3 expression in 32 clinical patients with breast cancer was positively correlated with lower survival probability, whereas lower GRIK3 expression in 20 clinical patients with breast cancer showed a negative correlation (https://precog.stanford.edu/; Figure 1F). Taken together, these results suggested that the mechanism underlying how *GRIK3* was transcribed into mRNA and further translated into protein is complicated and requires further study, however GRIK3 expression at protein level could be served as a biomarker for breast cancer.

### 3.2 GRIK3 promotes breast cancer cell proliferation and migration

**Figure 2:**
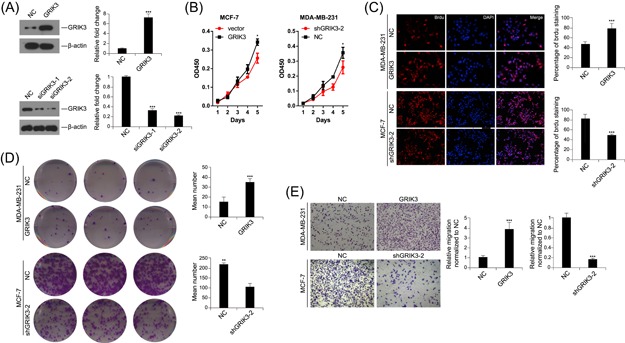
GRIK3 promoted cell proliferation and migration. A, Western blot analysis of GRIK3 expression in GRIK3 overexpression and knockdown cells. ***P < 0.001 by One‐way ANOVA. B, The CCK‐8 assay showing the effect of GRIK3 overexpression or knockdown on the proliferation of breast cancer cell lines. *P < 0.05 by the repeated measure of ANOVA. C. The number of positive cells was calculated in GRIK3 overexpressing cells, GRIK3 knockdown cells and the control cells in the BrdU assay. ***P < 0.001 by One‐way ANOVA. D, The colony formation assay was used to detect the effect of GRIK3 overexpression or knockdown on MDA‐MB‐231 proliferation. **P < 0.01 and ***P < 0.001 by One‐way ANOVA. E, Migration ability of breast cancer cells induced by GRIK3 overexpression or knockdown was determined by transwell migration assay. ***P < 0.001 by one‐way ANOVA. ANOVA, analysis of variance; GRIK3, glutamate receptor ionotropic, kainate 3; mRNA, messenger RNA [Color figure can be viewed at wileyonlinelibrary.com]

Given the high expression level of GRIK3 in breast cancer tissues, we hypothesized that GRIK3 could be an oncogene. Heterogenous expression and knockdown of GRIK3 was generated in MCF‐7 and MDA‐MB‐231, respectively (Figure 2A). CCK‐8 assay showed that overexpression of GRIK3 could promote the proliferation abilities of MCF‐7, whereas knockdown of GRIK3 significantly inhibited MDA‐MB‐231 growth (Figure 2B). In the BrdU assay, the fluorescence staining was much stronger in GRIK3 overexpressing cells than that in the control cells. In contrast, knockdown of endogenous GRIK3 markedly decreased the growth of MDA‐MB‐231 (Figure 2C). The colony formation assay showed that overexpression of GRIK3 significantly promoted the colony formation ability of MDA‐MB‐231 and GRIK3 knockdown exhibited a reduced number of colony formation (Figure 2D). To investigate further whether GRIK3 plays a role in cell migration, we performed a transwell migration assay. Compared with the control group, the number of cells that migrated through the polycarbonate membrane was significantly increased in the GRIK3 overexpressing group. On the other hand, knockdown of GRIK3 in MDA‐MB‐231 reduced cell migration (Figure 2E). These results indicated that GRIK3 could induce in vitro cell proliferation and migration.

### 3.3 GRIK3 promotes the growth of subcutaneously transplanted tumors in nude mice

**Figure 3:**
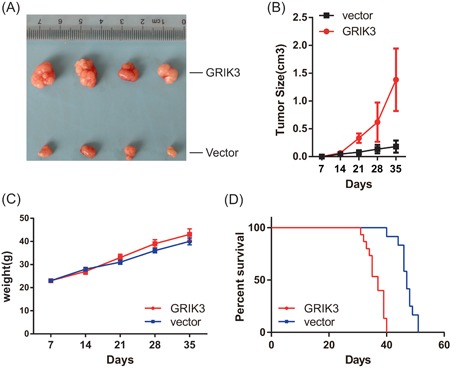
GRIK3 promoted the growth of the subcutaneous xenotransplanted breast tumors. A, Representative pictures of the resected tumors from mice injected with GRIK3 overexpressing cells or the control cells. B, Statistics of the tumor size at different time points. C, Measurement of the weight of nude mice in different time points. D, The survival curve between the groups of mice with or without GRIK3 overexpression. GRIK3, glutamate receptor ionotropic, kainate 3 [Color figure can be viewed at wileyonlinelibrary.com]

To investigate whether GRIK3 could promote tumor growth in nude mice, 1 × 10^6^ MDA‐MB‐231 GRIK3 overexpressing cells and corresponding control cells were injected subcutaneously. The tumor size and the weight of each nude mouse were measured every week. After 5 weeks, the tumors were surgically removed, photographed, and weighed. The results showed that, compared with the control group, overexpression of GRIK3 significantly promoted the growth of tumors (Figure 3A and 3B). The mice injected with GRIK3 overexpressing cells had survival times much shorter than the control group (Figure 3D). However, there were no significant differences in the weight between the two groups (Figure 3C). On the basis of these results, we concluded that GRIK3 is involved in the tumorigenesis of breast cancer.

### 3.4 The transcriptomic analysis and the genes expressed differentially between the GRIK3 overexpressing cells and the control cells

**Figure 4:**
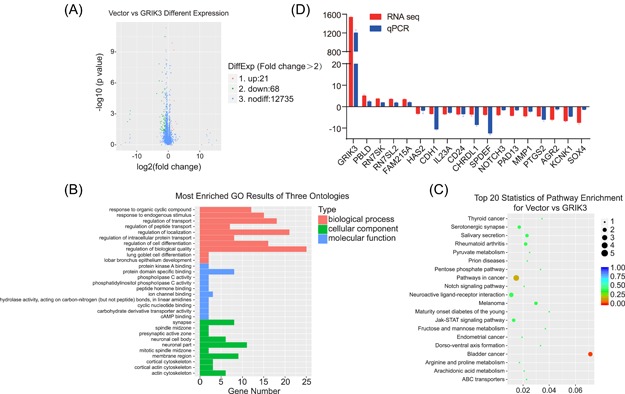
The gene expression profile and key signal pathways regulated by GRIK3. A, The volcano plot of the differential genes. B, GO analysis of the differential genes. C, KEGG analysis of the differential genes. D, Validation of the differentially expressed genes by qPCR. GO, gene ontology; GRIK3, lutamate receptor ionotropic, kainate 3 [Color figure can be viewed at wileyonlinelibrary.com]

To explore further the molecular mechanisms underlying GRIK3 related signaling, we compared the transcriptional landscape between GRIK3 overexpressing cells and the control cells using RNA‐sequencing (Figure 4A). Total RNA were extracted from GRIK3 overexpressing cells and control cells and subsequently sequenced. Comparing with the control group (fold change >2), a total of 21 genes showed higher expression levels in GRIK3 overexpressing cells than in the control cells. Meanwhile, 68 genes were downregulated and the expression of 12 735 genes did not change significantly. The GO analysis of the differentially expressed genes showed that GRIK3 mainly influenced the biological process “regulation of biological quality” and neuronal part of the molecular function (Figure 4B). Interestingly, GRIK3 was also involved in actin cytoskeleton regulation, indicating a possible role of GRIK3 in the morphologic change of tumor cells. The KEGG analysis revealed that GRIK3 affected several key signaling pathways in breast cancer, such as the Notch signaling pathway and Jak‐STAT signaling pathway (Figure 4C). We then verified the mRNA expression of 18 genes with | fold change | ≥ 3 using quantitative real‐time PCR (qPCR). Up‐ or downregulation of the average fold change for the genes were correlated with that in the sequencing results (Figure 4D). Among the 18 genes, CDH1, a classical epithelial biomarker, and its transcriptional factor SPDEF were both downregulated with the highest fold change among the validated genes in the qPCR assay. Therefore, we focused on the direction of the following mechanism study on the SPDEF/CDH1 pathway.

### 3.5 SPDEF and CDH1 mediated the cell proliferation, cell migration, and invasion abilities enhanced by GRIK3

**Figure 5:**
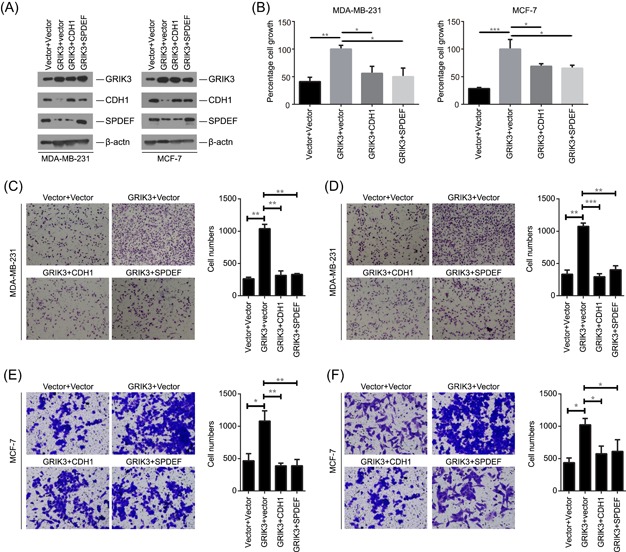
CDH1 and SPDEF inhibited cell proliferation, migration and invasion abilities mediated enhanced by GRIK3. A, Western blot analysis of GRIK3, CDH1, and SPDEF in GRIK3 overexpressing cells that were transfected with CDH1, SPDEF, or vector, respectively. B, Th expression of CDH1 and SPDEF suppressed the cell proliferation abilities induced by GRIK3 overexpression in MDA‐MB‐231 and MCF‐7. C,D, The expression of CDH1 and SPDEF inhibited the cell migration and invasion abilities enhanced by GRIK3 in MDA‐MB‐231 cells. E,F, The expression of CDH1 and SPDEF inhibited the cell migration and invasion abilities enhanced by GRIK3 in MCF‐7 cells. SPDEF, SAM pointed domain containing ETS transcription factor [Color figure can be viewed at wileyonlinelibrary.com]

CDH1 and SPDEF are closely associated with cancer cell metastasis. To address whether the cell proliferation and migration abilities enhanced by GRIK3 depended on the downregulation of SPDEF/CDH1, CDH1 and SPDEF were transiently expressed in GRIK3 stably expressing cell lines, MDA‐MB‐231 and MCF‐7, respectively (Figure 5A). The ability of GRIK3‐induced cell proliferation was significantly suppressed by the restoration of CDH1 or SPDEF expression (Figure 5B). Moreover, the enhanced migration and invasion activities by GRIK3 were also decreased upon the restoration of CDH1 or SPDEF expression in both MDA‐MB‐231 and MCF‐7 cells (Figure 5C‐F). Taken together, these results indicate that SPDEF/CDH1 signaling is required for the GRIK3‐mediated cell proliferation, migration, and invasion of breast cancer cells.

### 3.6 GRIK3 overexpression induced epithelial‐mesenchymal transition

**Figure 6:**
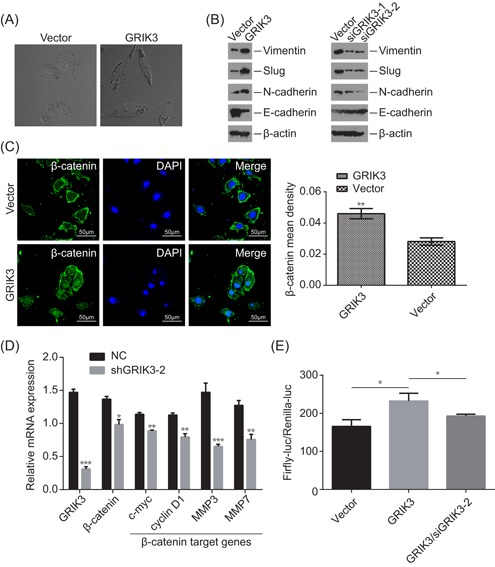
Overexpression of GRIK3 promoted EMT. A, Phase‐contrast microscopic images of GRIK3 overexpressing cells and the control cells. B, Western blot showing the effect of GRIK3 overexpression and knockdown on the expression of epithelial and mesenchymal markers in GRIK3 overexpressing cells and the control cells. This assay was repeated in triplicate. C, The expression of β‐catenin levels between GRIK3 overexpressing cells and the control cells was observed under a confocal microscope. **P < 0.01 D, qPCR assay measuring the mRNA expression of β‐catenin downstream target gene in response to GRIK3 overexpression. *P < 0.05, **P < 0.01, and ***P < 0.001. E, Relative luciferase activity promoted by β‐catenin promoter was measured in control cells, GRIK3 overexpressing cells and GRIK3 overexpressing cells that transfected with siGRIK3. Luc: luciferase. *P < 0.05 by one‐way ANOVA. ANOVA, analysis of variance; EMT, epithelial‐mesenchymal transition; GRIK3, glutamate receptor ionotropic, kainate 3; mRNA, messenger RNA [Color figure can be viewed at wileyonlinelibrary.com]

As the SPDEF/CDH1 pathway is involved in mechanisms regulating cell‐cell adhesions, mobility, and proliferation of epithelial cells and suppressed breast cancer metastasis through epithelial‐mesenchymal transition (EMT), we hypothesized that GRIK3 might play an important role in EMT. Phase‐contrast microscopy showed that the GRIK3‐overexpressing cells lost cell‐cell contacts and had spindle‐shaped morphology, which are hallmarks of cellular morphologic changes during EMT (Figure 6A). Immunoblot assays demonstrated that E‐cadherin, the epithelial markers, were inhibited, whereas mesenchymal markers, such as Vimentin, Slug, and N‐cadherin were upregulated in GRIK3‐overexpressing cells (Figure 6B). In contrast, knockdown of GRIK3 reduced the expression of Vimentin, Slug, and N‐cadherin and increased E‐cadherin expression (Figure 6B). Interestingly, the same tendency of protein expression changes of E‐cadherin, Vimentin, Slug, and N‐cadherin accorded with their mRNA expression changes, according to the RNA‐seq data of GRIK3 overexpressing cells and control cells. Using immunofluorescence to visualize intracellular β‐catenin, we observed an increase in β‐catenin levels and the translocation of β‐catenin from cell membrane to nucleus (Figure 6C). To validate whether the translocation of β‐catenin is the key step for its transcriptional activation, we examined several downstream target genes of β‐catenin. As shown in Figure 6D, the mRNA levels of *c‐myc*, *cyclin D1*, *MMP3,* and *MMP7* were upregulated in response to GRIK3 overexpression. Furthermore, overexpression of GRIK3 significantly enhanced the luciferase activity driven by β‐catenin promoter, whereas the rescue assay showed that knockdown of GRIK3 by siRNA in GRIK3 overexpressing cells decreased β‐catenin promoter activity. These results indicated that the expression of GRIK3 could promote EMT initiation and progression partly by β‐catenin activation.

## 4 DISCUSSION

Mounting research has shown that many subtypes of the glutamate receptor family are important in the central nervous system and neurologic tumors.^12^, ^13^, ^14^ However, whether this group of proteins have significant effects in malignancies remains to be discovered. As a member of the KA‐R subfamily, our understanding of GRIK3 has hitherto been limited in its expression in different malignancies. Whether GRIK3 could switch some tumor phenotypes, such as cell proliferation, apoptosis, and metastasis, remains unclear. GRIK1, in the same family as GRIK3, is a novel susceptibility gene for HBV‐related HCC, and was revealed by a large three‐stage genome‐wide association study.^15^ GRIK2 is a tumor suppressor in gastric cancer. DNA hypermethylation of the GRIK2 promoter inhibited its expression and decreased cell migration capabilities.^16^ These studies indicated that members of KA‐R family play different roles in different malignancies. In this study, we provided evidence that the expression of GRIK3 is upregulated in breast cancer. GRIK3 promotes cell proliferation and migration in vitro and tumor growth in vivo. Mechanically, GRIK3 could facilitate EMT through inhibiting the epithelial marker, CDH1, and its transcription factor SPDEF, while increasing the expression of the mesenchymal marker, such as ZEB1 and β‐catenin.

SAM pointed domain containing ETS transcription factor (SPDEF), also known as prostate‐derived ETS factor, belongs to the ETS (E26 transformation specific) transcription factor family and is a specific transcription factor which preferentially binds a GGAT DNA motif rather than the GGAA ETS family consensus sequence.. The expression of SPDEF could be detected in multiple organs, including the airway, breast, gastric, prostate, and small and large intestinal epithelia, and is able to inhibit the cell proliferation.^17^ It has also been demonstrated that SPDEF acts as a tumor and metastasis suppressor in multiple epithelium‐derived cancers.^18^ For example, SPDEF inhibits survival of prostate cancer cells through the androgen receptor (AR) relevant for cancer signaling, suppresses colorectal cancer through the E‐cadherin and β‐catenin pathway,^18^ and modulates epithelial‐mesenchymal‐transition (EMT) to lower migration and invasion abilities of bladder carcinoma cells by upregulating E‐cadherin expression and downregulating the expression of N‐cadherin, SNAIL, SLUG, and vimentin.^19^ SPDEF is an important molecule for driving invasive mucinous adenocarcinoma of the lung (IMA) transformation.^20^

E‐cadherin (CDH1) is a type I transmembrane protein, mainly expressed in epithelial cells where it plays a vital role in maintaining cell polarity and integrity.^21^ In tumors, it functions as a powerful molecular barrier that inhibits cancer cell proliferation, shedding, invasion, and metastasis.^21^ The absence of functional E‐cadherin possibly caused by truncating mutations, loss of heterozygosity, and transcriptional repression is causal for invasive lobular breast carcinoma, which is a subtype of breast cancer.^22^ In addition, it has shown that E‐cadherin improves IGF1R recruitment to adherens junctions, possibly resulting in receptor sequestration and signaling repression. The loss of functional E‐cadherin would impair the adherens junction formation, and release IGF1R to re‐localize to the entirety of the cell membrane where the IGF1 ligand is easily bound for initiating cancer signaling.^10^

The present study is the first study suggesting that GRIK3 is an oncogene in breast cancer and is involved in the EMT pathway. Although the biological functions of GRIK3 in breast cancer have been exposed only slightly, additional research should be conducted into the mechanics of its oncogenic effects, such as how GRIK3 inhibits the expression of CDH1 and is the structure of the GRIK3‐centric protein interaction network. A greater understanding of membrane protein GRIK3 offers new opportunities for the design of anti‐GRIK3 therapeutic strategies.

## CONFLICT OF INTERESTS

The authors declare that there are no conflict of interests.

## AUTHOR CONTRIBUTIONS

LHL and ZHS conceived and designed the study. LT, HYY, DC, WWL, JRL, and QZ performed the experiments. BX and ZZK analyzed the data and wrote the manuscript. All authors have read and approved the final manuscript.
